# Does Ultrasound Guidance Improve the Effectiveness of Neurotoxin Injections in Patients with Cervical Dystonia? (A Prospective, Partially-Blinded, Clinical Study)

**DOI:** 10.3390/toxins14100674

**Published:** 2022-09-28

**Authors:** Małgorzata Tyślerowicz, Jarosław Dulski, Justyna Gawryluk, Jarosław Sławek

**Affiliations:** 1 Department of Neurophysiology, Copernicus Memorial Hospital, 93-513 Lodz, Poland; 2 Department of Neurology, Mayo Clinic, 4500 San Pablo Rd., Jacksonville, FL 32224, USA; 3 Department of Neurology, St. Adalbert Hospital, Copernicus PL Ltd., 80-462 Gdansk, Poland; 4 Division of Neurological and Psychiatric Nursing, Faculty of Health Sciences, Medical University of Gdansk, 80-211 Gdansk, Poland; 5 Department of Neurology, Faculty of Medical Sciences in Katowice, Medical University of Silesia, 41-808 Katowice, Poland

**Keywords:** ultrasound guidance, cervical dystonia, botulinum neurotoxin, efficacy

## Abstract

Aim: The aim of this study was to evaluate the efficacy of ultrasound guidance (US) in the treatment of cervical dystonia (CD) with botulinum neurotoxin type A (BoNT-A) injections in comparison to anatomical landmarks (AL). To date, US is routinely used in many centers, but others deny its usefulness. Materials and Methods: Thirty-five patients (12 males, 23 females) with a clinical diagnosis of CD were included in the study. Intramuscular administration of BoNT-A was performed using either US guidance, or with AL, in two separate therapeutic sessions. The efficacy of BoNT-A administration was assessed with the Toronto Western Spasmodic Torticollis Rating Scale (TWSTRS), Tsui modified scale, Craniocervical Dystonia Questionnaire (CDQ-24) and Clinical Global Impression—Improvement scale (CGI-I). Additionally, patients at therapeutic sessions were digitally recorded and evaluated by two blinded and independent raters. Results: A significant decrease in total TWSTRS, severity subscale TWSTRS, Tsui score, and CDQ-24 was found in both the AL and US group; however, in the TWSTRS disability and pain subscales, a significant decrease was found only in the US group. Moreover, US guided treatment also resulted in a greater decrease in TWSTRS, Tsui score and CDQ-24 compared to anatomical landmarks use only. Conclusions: US guidance might be helpful in improving the results of BoNT-A injections in cervical dystonia, reducing associated pain and disability; however, more studies are needed to evaluate its clinical efficacy.

## 1. Introduction

Cervical dystonia (CD) is the most common form of focal dystonia characterized by sustained, involuntary or intermittent muscle contractions and/or twitching resulting in abnormal postures and/or positioning of the head and/or neck [1]. Botulinum neurotoxin (BoNT) is recommended as the first line treatment for CD [2,3], with statistically significant improvement in clinical studies in 70–90% of patients [4,5]. In everyday practice many patients experience an improvement; nevertheless, it is suboptimal in a majority of them [6,7]. This may be caused by several factors including inaccurate diagnosis, primary or secondary resistance to treatment and invalid drug storage. Nevertheless, it seems that appropriate pattern classification, a physician’s experience, BoNT-A dose and precision of injections are the most important factors contributing to successful treatment of CD [8,9].

Initially, CD was classified into four types, related to: turning the head (torticollis), tilting the head to one side (laterocollis), backwards (retrocollis), or forwards (anterocollis) [10]. The Col-Cap concept, introduced by Reichel et al. [11] in consonance with anatomic and imaging studies, identified 10 main types of CD. According to this concept, deeply located muscles like obliquus capitis inferior (OCI), semispinalis cervicis or longus colli are involved. Jost et al. [12] confirmed that during the last several decades some new muscles, which were previously never, or hardly ever, considered, have been added. Based on this study the most common injected muscles besides splenius capitis, sternocleidomastoid (scm) and trapezius were the levator scapulae, semispinalis capitis and OCI. It seems to be that deep lying muscles are difficult to be located and precisely injected without any visualization. Muscles can be injected using anatomical landmarks, under electromyography (EMG), computed tomography (CT) or ultrasound (US) guidance, but it remains controversial whether EMG and/or US are helpful in clinical practice. There are studies that support the role of EMG indicating that injections performed using only anatomic landmarks are unreliable (83% reached sternocleidomastoid, but only 47% the levator scapulae muscle) [13]. The other study evaluating the accuracy of anatomy-guided injections also showed that even targeting first-layer muscles can be difficult (splenius capitis 67.9%, scm 86.7%, trapezius 75%, levator scapulae 78.3% of accuracy) [14].

Ultrasound-guided injection provides real-time visualization of muscles and adjacent anatomical structures, which eventually may result in more precise injections and potentially a lower number of side effects [15]. Nevertheless, thus far there is a lack of research elucidating the real effectiveness of US-guided treatment.

Most of the studies have been performed in small groups of patients or were focused on injections into deep cervical muscles only [16,17,18,19,20,21]. There is only one recently published study directly comparing the results of US-guided and non-guided BoNT-A injections in two groups of patients [22]. Nevertheless, there are no randomized, controlled studies proving the higher effectiveness of US-guided versus blinded injections. In order to address this hypothesis, the aim of our study was to evaluate the efficacy of US-guided BoNT-A injections in comparison with using anatomic landmarks.

## 2. Results

**Figure 1:**
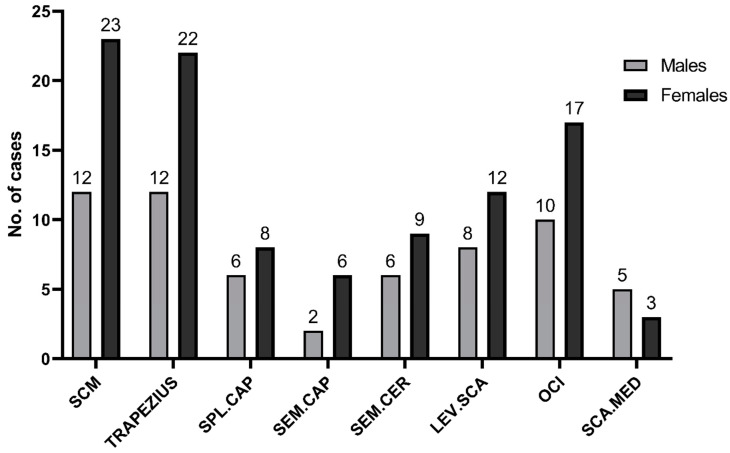
No. of muscles that were injected with botulinum toxin. SCM—m.sternocleidomastoideus. SPL.CAP—m.splenius capitis. SEM.CAP—m.semispinalis capitis. SEM.CER—m.semispinalis cervicis. LEV.SCA—m.levator scapulae. OCI—m.obliquus capitis inferior. SCA.MED—m.scalenus medius.

The mean disease duration was 9.43 ± 2.93 years and mean treatment time was 43 ± 29 months. The majority of study participants (*n* = 28, 80%) were diagnosed with complex type of CD (45.7% torticaput combined with laterocaput, 11.4% torticaput with laterocollis, 8.5% lateral shift, 8.5% anterior saggital shift and 5.7% retrocaput with laterocaput). Twenty percent of patients presented pure torticaput. Muscles that were injected are shown in Figure 1. All of the participants were previously treated with BoNT-A, but none were treated according to the Col-Cap concept with US-guided injections. Twenty percent of patients (*n* = 7) used baclofen occasionally and 28.5% (*n* = 10) of patients were diagnosed with depression and were treated with SSRIs (selective serotonin reuptake inhibitors). Detailed characteristics of the study group are presented in Table 1.

### 2.1. Clinical Assessments

**Figure 2:**
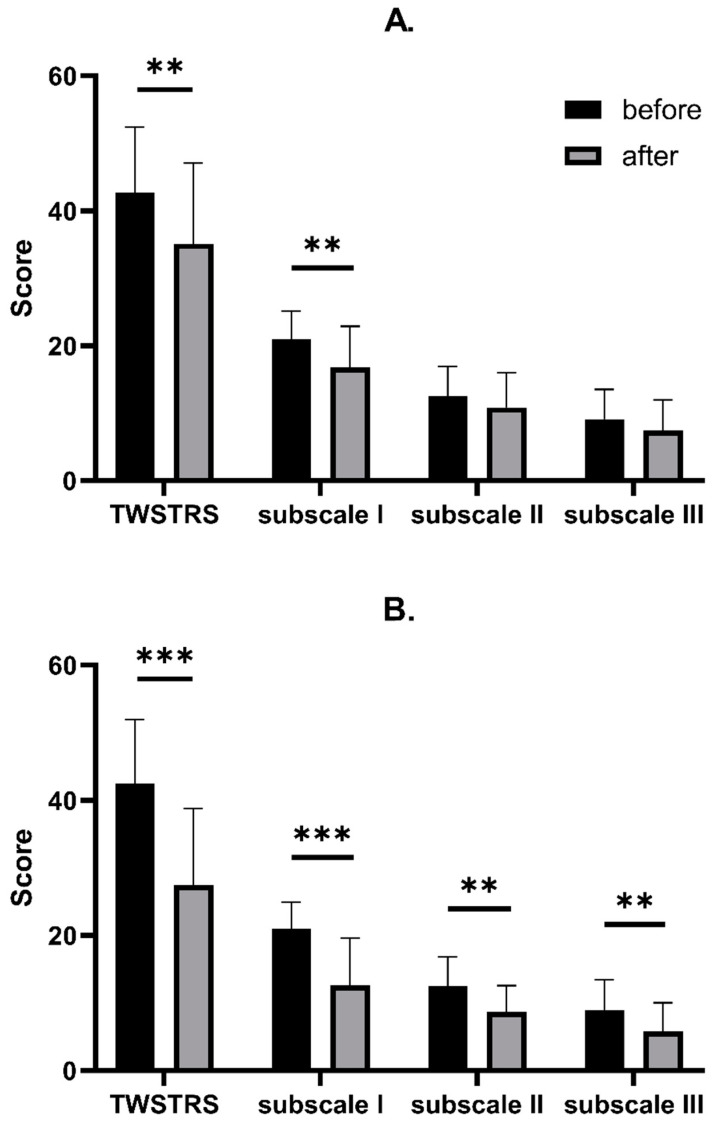
Comparison between TWSTRS decrease after BoNT-A injection with AL (**A**) and with US (**B**). Asterisks indicate the level of statistical significance (** *p* < 0.001, *** *p* < 0.0001).

**Figure 3:**
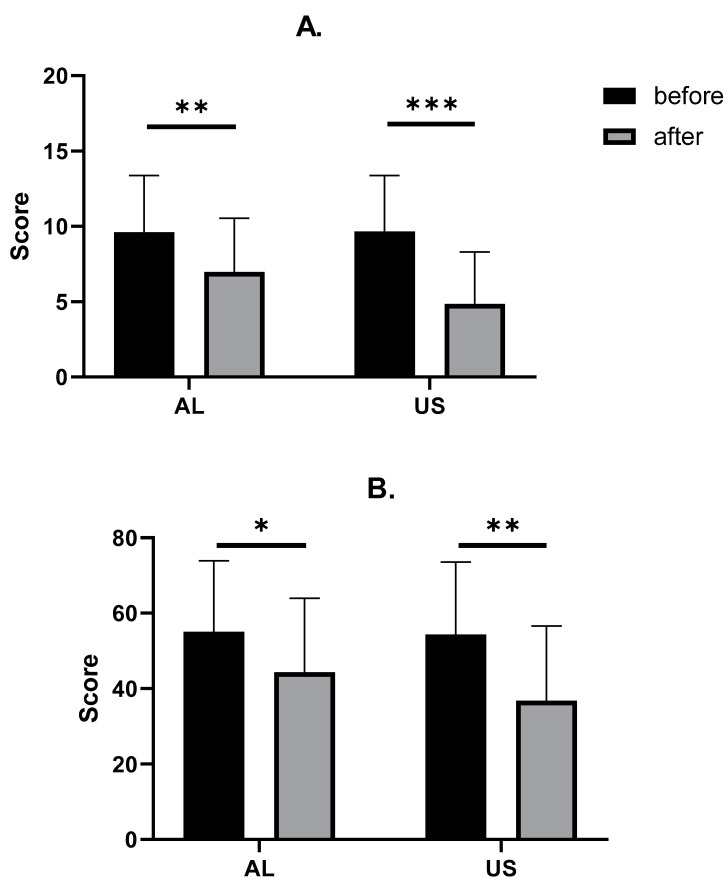
Comparison between TSUI (**A**) and CDQ-24 (**B**) changes after BoNT-A injections. Asterisks indicate the level of statistical significance (* *p* < 0.05, ** *p* < 0.001, *** *p* < 0.0001).

**Figure 4:**
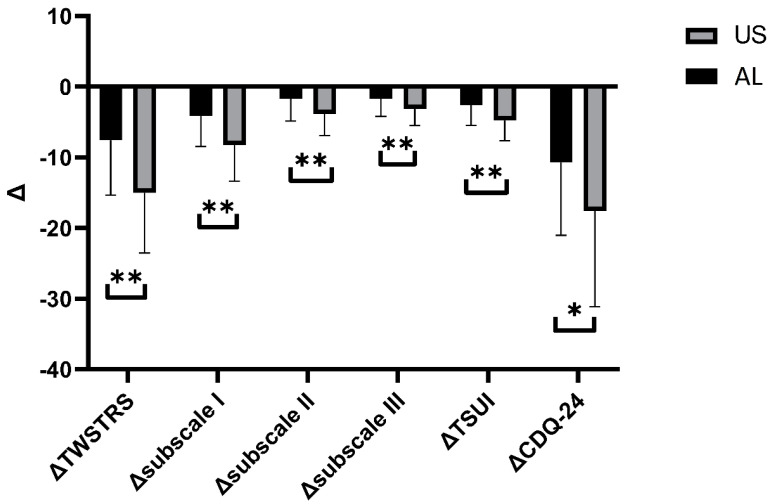
Comparison between Δvalues for each variable. Asterisks indicate the level of statistical significance (* *p* < 0.05, ** *p* < 0.001).

A significant decrease in total TWSTRS score was found both in the AL (from 42.71 ± 9.67 to 35.11 ± 11.97, *p* = 0.0047) and the US (from 42.49 ± 9.50 to 27.46 ± 11.34, *p* < 0.0001) groups. Detailed analysis of TWSTRS subscales revealed that there was a significant decrease in TWSTRS severity subscale in AL (from 20.94 ± 4.22 to 16.77 ± 6.12, *p* = 0.0015) and US (from 20.94 ± 3.94 to 12.66 ± 6.92, *p* < 0.0001); however, in the TWSTRS disability and pain subscales, a significant decrease was found only in the US group (from 12.54 ± 4.27 to 8.69 ± 3.9, *p* = 0.0002, and from 9.00 ± 4.47 to 5.83 ± 4.23, *p* = 0.0033, respectively). We also found a significant decrease in Tsui scores in both the AL (from 9.60 ± 3.77 to 6.97 ± 3.57, *p* = 0.0038) and US groups (from 9.66 ± 3.72 of 4.86 ± 3.46, *p* < 0.0001). Finally, a significant decrease in the CDQ-24 score was observed both in AL (from 55.09 ± 18.84 to 44.34 ± 19.63, *p* = 0.0224) and US (from 54.34 ± 19.19 to 36.74 ± 19.89, *p* = 0.0003). Detailed results are shown in Table 2 and Figure 2 and Figure 3. To study the exact effect of US use on a patient’s clinical improvement, we decided to subtract the results of measurements over time (value_after_ − value_before_) and create a Δvalue. Use of US significantly decreased the scores in all studied scales (TWSTRS, Tsui score, CDQ-24) compared to the AL group. Detailed results are shown in Table 3 and Figure 4.

### 2.2. Correlations between Clinical Scales

Next, we aimed to determine whether the change in TWSTRS total score (ΔTWSTRS) and severity subscale (ΔTWSTRS) score correlates with TSUI (ΔTSUI). We found a strong correlation between ΔTWSTRS severity subscale and ΔTSUI in the AL group (ρ = 0.72, *p* < 0.0001) and moderate correlations between ΔTWSTRS and ΔTSUI in both the AL and US groups (ρ = 0.49, *p* = 0.0027, and ρ = 0.49, *p* = 0.0030, respectively, data you can find in a supplementary File S2.

### 2.3. Blinded Raters’ Assessments

**Figure 5:**
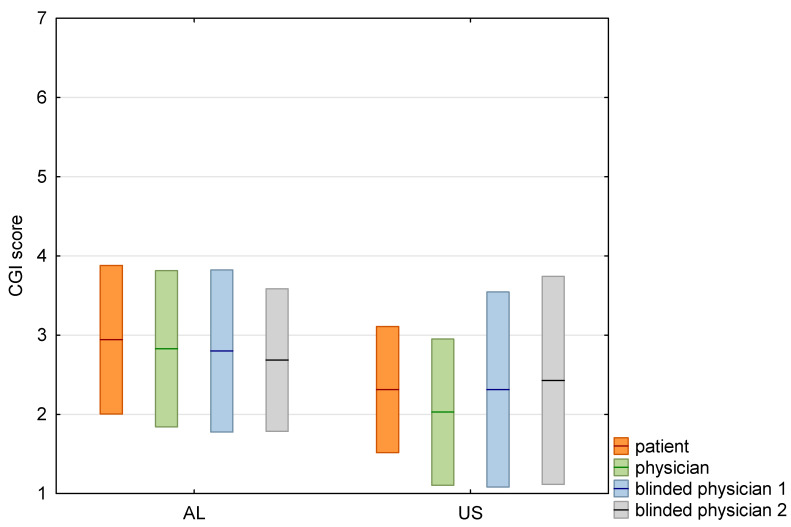
Box-plot with comparison of mean CGI-I scores graded by patient, physician and two blinded physicians. Means shown as lines, standard deviations as boxes.

In the last part of the analysis, we have evaluated the blinded raters’ assessments according to CGI-I score. Treated patients and two blinded experts (No1, No2) assessed treatment effects versus the study physician. For AL and US injections, we observed significant correlations between study physician and patient (ρ = 0.53), and blinded physicians No1 (ρ = 0.72) and No2 (ρ = 0.68), and between study physician and patient (ρ = 0.60), and blinded physicians No1. (ρ = 0.82) and No2. (ρ = 0.66), respectively. Detailed results are shown in Table 4 and Table 5, and in Figure 5.

### 2.4. Incidence of Side Effects

Additionally, we calculated side effects after both types of treatment (AL and US). We found no significant differences between the incidence of side effects, e.g., swallowing problems, pain at the site of injection and head drooping, between compared groups. Detailed results are shown in Table 6. No other treatment related adverse events were reported.

## 3. Discussion

The novel approach to BoNT-A treatment of CD include the identification of dystonia pattern according to the Col-Cap concept [23]. According to this concept, in many forms of CD deeply located muscles, which are difficult to identify without imaging, like OCI or semispinalis cervicis, are involved. On the other hand, superficial muscles (like trapezius, scm, splenius capitis) also may be easily missed without any visualization [13,14]. In studies comparing the blinded (AL) and EMG guided/verified [13] or US verified injections [14], even in the hands of an experienced physician there was a substantial rate of missed muscles.

Ultrasonography is a convenient, non-invasive method that enables the visualization of muscles and surrounding structures, including nerve bundles and large vessels, in real time. It is hypothesized that using US increases the accuracy of injections of both superficial and deep cervical muscles. Available data on using US in the treatment of CD is scarce. Bhidayasiri et al. [16] presented a case series of three patients about whom they conclude the lack of deeply located muscles imaging led to BoNT-A treatment failure. There is only one study comparing directly US-guided and non-guided injections in two different groups of patients which found no difference [22]. An expert-statement published in 2015 [24] suggested that US-guided injections should be used especially in cases with specific anatomic conditions, such as pronounced or inaccessible neck muscles, obesity or muscle atrophy, during adverse events following BoNT-A treatment, complex dystonic patterns with involvement of deep cervical muscles, or in secondary non-responders. Nevertheless, evidence is still lacking for proving the real effectiveness of US-guided vs. AL injections in CD treatment. In our study, the response to treatment measured by clinical scales was significant in both US-guided and AL injections, but a greater decrease in scales was associated with US use. The consistency of the results obtained by unblinded and blinded raters proved this hypothesis. Despite the effectiveness of both, the comparison of the two approaches (AL vs. US) showed the superiority of US-guided injections (Table 3). All injections and US assessments were performed by one and the same experienced (15 years) and certified neurologist, which is why it cannot be excluded that the results in group AL were influenced by the principal investigator’s knowledge of anatomy and experience in the use of ultrasound.

Hong et al. [15] revealed that use of US during injections decreases the incidence of dysphagia (0% vs. 34.7%), but no exact conclusions have been made due to the small number of participants (*n* = 5). Similarly, in our study, swallowing problems were less frequent in the US-guided group, but the difference did not reach significance. In our opinion, a low number of patients may have been relevant. On the other hand, a retrospective study on a group of 75 CD patients revealed that US guidance is not able to prevent dysphagia [25].

In summary, US can be helpful in improving the results of BoNT-A injections in CD.

There are, however, several limitations of our study. First of all, we have not employed a randomized trial protocol, nor a case-control study. The study was only partially blinded. The assessments based on TWSTRS and Tsui scores were not blind, with the only blind assessment being the CGI assessment. To study the exact effect of US use on a patient’s clinical improvement, we decided to subtract the results of measurements over time (value_after_ − value_before_) and create a Δvalue which showed a greater decrease in scales, but more studies are needed to evaluate whether it has clinically relevant effect. On the other hand, we compared the results of AL and US in the same patients, which seems to be a more reliable method, taking into account the high variability of CD patterns. Our approach does not provide information about the possible influence of other variables (e.g., sex, age, disease duration, BoNT-A preparation and doses) on treatment efficacy. However, comparing the results in one patient allowed us to exclude the influence of many factors (e.g., dose or dystonia pattern, co-morbidities like depression or anxiety) which could influence the overall effect [5,26,27].

To the best of our knowledge, this is one of the most complex studies performed to date, showing that US-guided injections might be helpful in improving the results of BoNT-A injections in cervical dystonia. We definitely need more larger and controlled studies to prove the concept.

## 4. Conclusions

US guidance might be helpful in improving the results of BoNT-A injections in cervical dystonia, reducing associated pain and disability; however, more studies are needed to evaluate its clinical efficacy.

## 5. Materials and Methods

### 5.1. Study Design and Patients

Thirty-five consecutive patients (12 males, 23 females, mean age 52.49 ± 10.05) with clinical diagnosis of idiopathic CD were recruited from the outpatient clinic from the group who participate in the routine therapeutic program for cervical dystonia. They were the patients who fulfilled the following inclusion criteria: ≥18 years of age, previous effective treatment with BoNT-A, the recurrence of symptoms and at least 12 weeks interval after the last injection. The study was prospective and each patient underwent two sessions: the first one in which injections were administered according to anatomic landmarks (AL group) and, after no less than 12 weeks, the second one was performed under US guidance (US group). The second session was only performed on condition that the patient had returned to the clinical condition from before the first session (the same number of points in the scales). Patients were evaluated by the same physician (the so-called Study Physician) with 15 years of experience who performed a clinical examination and classified dystonic posture on the basis of the Col-Cap concept [23,28].

The choice of muscles, type of BoNT-A, total dose and each muscle dose remained the same as before and during both sessions. Each session was digitally video recorded according to the same protocol.

In US-guided injection, we used a Hitachi Arietta 50 device with a linear probe (6–13 MHz) and out-of-plane approach.

The landmarks used for injections and muscle injection sites with US use are presented in the Supplementary Materials.

All patients signed informed consent before enrollment into the study. The study was approved by the local bioethics committee (Nr of consent K.B.-2/17).

### 5.2. Assessments

All patients were evaluated before each session and 4 weeks after the injection. The efficacy assessments included Toronto Western Spasmodic Torticollis Rating Scale (TWSTRS, including total scores, severity, disability and pain subscale scores); Tsui-modified score; Craniocervical Dystonia Questionnaire (CDQ-24); and Clinical Global Impression–Improvement (CGI-I) score (calculated on a scale from 1-very much improved to 7-very much worse). CGI-I was assessed by patients, injector and two independent blinded raters to whom standardized video recordings were sent. The patients were filmed in frontal position (with open and closed eyes, and with shoulder stabilization), from the side, and while walking. Both raters were experienced neurologists with at least five years of experience in BoNT-A treatment of CD. Safety assessments included incidence of adverse events: dysphagia, muscle pain and head drop as obligatory questions and others spontaneously reported by patients.

### 5.3. Statistical Analysis

Statistical analysis was performed using GraphPad Prism 9 and Statistica 13 software. Continuous variables were presented using mean and standard deviation, while numerical and non-continuous variables as number of cases (*n*) and percentage, median and interquartile range. Distribution of the variables was assessed using the Shapiro–Wilk test. To compare differences between groups, the Student’s *t*-test in Welch’s modification or the Mann–Whitney U test were used. The correlation was performed using the Spearman correlation coefficient. A correlation coefficient ranging from 0.00 to 0.19 was considered as very weak, 0.20 to 0.39 as weak, 0.40 to 0.59 as moderate, 0.60 to 0.79 as strong and 0.80 to 1.0 as very strong. A *p* value below 0.05 was deemed significant.

## Figures and Tables

**Table 1:** Baseline characteristics of the study group.

	Study Group (*n* = 35)	Males (*n* = 12)	Females (*n* = 23)
Age (years) at the time of study	52.49 ± 10.05	50.00 ± 12.88	53.78 ± 8.23
Treatment time (prior the study)	43.06 ± 28.96	56.92 ± 33.05	35.83 ± 24.30
Disease duration (years)	9.43 ± 2.93	10.28 ± 3.24	8.98 ± 2.73
Type of BoNT-A and doses			
AbobotulinumtoxinA		11 (500–1000 U, mean 863.6 U)	12 (750 U)
OnabotulinumtoxinA		0	7 (200 U)
IncobotulinumtoxinA		1 (200 U)	4 (200 U)

**Table 2:** Comparison of mean scoring values for AL and US groups.

Scoring	AL			US		
	Before	After	*p*-Value	Before	After	*p*-Value
TWSTRS	42.71 ± 9.67	35.11 ± 11.97	0.0047	42.49 ± 9.50	27.46 ± 11.34	<0.0001
severity subscale	20.94 ± 4.22	16.77 ± 6.12	0.0015	20.94 ± 3.94	12.66 ± 6.92	<0.0001
disability subscale	12.57 ± 4.39	10.86 ± 5.11	0.1368	12.54 ± 4.27	8.69 ± 3.93	0.0002
pain subscale	9.11 ± 4.42	7.43 ± 4.58	0.1222	9.00 ± 4.47	5.83 ± 4.23	0.0033
Modified TSUI scale	9.60 ± 3.77	6.97 ± 3.57	0.0038	9.66 ± 3.72	4.86 ± 3.46	<0.0001
CDQ-24	55.09 ± 18.84	44.34 ± 19.63	0.0224	54.34 ± 19.19	36.74 ± 19.89	0.0003

**Table 3:** Comparison of mean change (Δ) for all scales.

Score	AL	US	*p*-Value
ΔTWSTRS total	−7.60 ± 7.76	-15.03 ± 15.51	0.0003
Δseverity subscale	−4.17 ± 4.29	-8.29 ± 8.09	0.0007
Δdisability subscale	−1.71 ± 1.15	-3.86 ± 3.05	0.0024
Δpain subscale	−1.69 ± 1.52	-3.17 ± 3.35	0.0084
ΔModified Tsui scale	−2.63 ± 2.85	-4.80 ± 4.86	0.0008
ΔCDQ-24	−10.74 ± 10.28	-17.60 ± 17.51	0.0185

**Table 4:** Averages of CGI scale scores by patient, physician, and experts 1 and 2 according to ultrasound use.

CGI-I	AL	US	*p*-Value
Patient	2.94 ± 0.94	2.31 ± 0.80	0.0069
Physician	2.83 ± 0.98	2.03 ± 0.92	0.0005
Blinded physician 1	2.80 ± 1.02	2.31 ± 1.23	0.0453
Blinded physician 2	2.69 ± 0.90	2.43 ± 1.31	0.0842

**Table 5:** Spearman correlation coefficients between raters and Study Physician in CGI-I scale.

	AL	US
Patient	ρ = 0.53; *p* = 0.001	ρ = 0.60; *p* = 0.001
Blinded physician 1	ρ = 0.72; *p* < 0.001	ρ = 0.82; *p* < 0.001
Blinded physician 2	ρ = 0.68; *p* < 0.001	ρ = 0.66; *p* < 0.001

**Table 6:** Incidence of side effects after BoNT-A administration.

	AL	US	*p*-Value
Swallowing problems	5 (14.29%)	1 (2.86%)	0.0877
Pain at the site of injection	10 (28.57%)	12 (34.29%)	0.6066
Head drop	2 (5.71%)	2 (5.71%)	-

## Supplementary Materials

The following supporting information can be downloaded at: www.mdpi.com/article/10.3390/toxins14100674/s1. File S1: The landmarks used for injections and muscle injection sites with US guidance; File S2: Spearman’s correlation coefficients between ΔTWSTRS and ΔTSUI in patients with AL (A) and with USG (B), and between ΔTWSTRS subscale I and ΔTSUI with AL (C) and with USG (D).

## References

[1] Brin M.F., Comella C., Brin M.F., Comella C., Jankovic J., Philadelphia P.A. (2004). Pathophysiology of dystonia. Dystonia: Etiology, Clinical Features, and Treatment.

[2] Simpson D.M., Blitzer A., Brashear A., Comella C., Dubinsky R., Hallett M., Jankovic J., Karp B., Ludlow C.L., Miyasaki J.M. (2008). Therapeutics and Technology Assessment Sucommittee of the American Acadamy of Neurology. Assessment: Botulinum neurotoxin for the treatment of movement disorders (an evidence-based review): Report of the Therapeutics and Technology Assessment Subcommittee of the American Academy of Neurology. Neurology.

[3] Albanese A., Asmus F., Bhatia K.P., Elia A.E., Elibol B., Filippini G., Gasser T., Krauss J.K., Nardocci N., Newton A. (2011). EFNS guidelines on diagnosis and treatment of primary dystonias. Eur. J. Neurol..

[4] Swope D., Barbano R. (2008). Treatment recommendations and practical applications of botulinum toxin treatment of cervical dystonia. Neurol Clin..

[5] Jankovic J., Adler C.H., Charles P.D., Comella C., Stacy M., Schwartz M., Sutch S.M., Brin M.F., Papapetropoulos S. (2011). Rationale and design of a prospective study: Cervical Dystonia Patient Registry for Observation of Ona Botulinumtoxin a Efficacy (CD PROBE). BMC Neurol..

[6] Comella C.L., Thompson P.D. (2006). Treatment of cervical dystonia with botulinum toxins. Eur. J. Neurol..

[7] Jinnah H.A., Goodmann E., Rosen A.R., Evatt M., Freeman A., Factor S. (2016). Botulinum toxin treatment failures in cervical dystonia: Causes, management and outcomes. J. Neurol..

[8] Tyślerowicz M., Kiedrzyńska W., Adamkiewicz B., Jost W.H., Sławek J. (2020). Cervical dystonia—Imroving the effectiveness of botulinum toxin therapy. Neurol. Neurochir. Pol..

[9] Sławek J., Jost W.H. (2021). Botulinum neurotoxin in cervical dystonia revisited—Recent advances and unanswered questions. Neurol. Neurochir. Pol..

[10] Jankovic J., Leder S., Warner D., Schwartz K. (1991). Cervical dystonia: Clinical findings and associated movement disorders. Neurology.

[11] Reichel G., Stenner A., Jahn A. (2009). The phenomenology of cervical dystonia Proposed New treatment strategy with botulinum toxin. Fortschr. Neurol. Psychiat..

[12] Jost W.H., Biering-Sorensen B., Drużdż A., Kreisler A., Pandey S., Sławek J., Tatu L. (2020). Preferred muscles in cervical dystonia. Neurol. Neurochir. Pol..

[13] Speelman J.D., Brans J.W. (1995). Cervical dystonia and botulinum treatment: Is electromyographic guidance necessary?. Mov. Disord..

[14] Kreisler A., Simonin C., Degardin A., Mutez E., Defebvre L. (2020). Anatomy-guided injections of botulinum neurotoxin in neck muscles: How accurate is needle placement?. Eur. J. Neurol..

[15] Hong J.S., Sathe G.G., Niyonkuru C., Munin M.C. (2012). Elimination of dysphagia using ultrasound guidance for botulinum toxin injections in cervical dystonia. Muscle Nerve.

[16] Bhidayasiri R. (2011). Treatment of complex cervical dystonia with botulinum toxin: Involvement of deep-cervical muscles may contribute to suboptimal responses. Parkinsonism Relat. Disord..

[17] Fujimoto H., Mezaki T., Yokoe M., Mochizuki H. (2012). Sonographic guidance provides a low-risk approach to the longus colli muscle. Mov. Disord..

[18] Sung D.H., Choi J.Y., Kim D.H., Kim E.S., Son Y.I., Cho Y.S., Lee S.J., Lee K.H., Kim B.T. (2007). Localization of dystonic muscles with 18F-FDG PET/CT in idiopathic cervical dystonia. J. Nucl. Med..

[19] Lee I.H., Yoon Y.C., Sung D.H., Kwon J.W., Jung J.Y. (2009). Initial experience with imaging-guided intramuscular botulinum toxin injection on patients with idiopathic cervical dystonia. AJR Am. J. Roentgenol..

[20] Tyślerowicz M., Jost W.H. (2019). Injection into the Longus Colli Muscle via the Thyroid Gland. Tremor Other Hyperkinet. Mov..

[21] Allison S.K., Odderson I.R. (2016). Ultrasound and Electromyography Guidance for Injection of the Longus Colli with Botulinum toxin for the Treatment of Cervical Dystonia. Ultrasound Q..

[22] Kreisler A., Djelad S., Simonin C., Baille G., Mutez E., Degardin A., Defebvre L., Labreuche J., Cailliau E., Duhamel A. (2022). Does ultrasound-guidance improve the outcome of botulinum toxin injections in cervical dystonia?. Rev. Neurol..

[23] Tatu L., Jost W.H. (2017). Anatomy and cervical dystonia: “Dysfunction follows form”. J. Neural. Transm..

[24] Schramm A., Baumer T., Fietzek U., Heitman S., Walter U., Jost W. (2015). Relevance of sonography for botulinum toxin treatment of cervical dystonia: An expert statement. J. Neural. Transm..

[25] Kutschenko A., Klietz M., Paracka L., Kollewe K., Schulte-Sutum A., Janssen T., Schrader C., Wegner F., Dressler D. (2020). Dysphagia in cervical dystonia patients receiving optimised botulinum toxin therapy: A single-center retrospective cohort study. J. Neural. Transm..

[26] Kessler K.R., Skutta M., Benecke R., Long-term treatmentof cervical dystonia with botulinum toxin A: Efficacy, safety, and antibody frequency (1999). German Dystonia Study Group. J. Neurol..

[27] Ferreira J.J., Colosimo C., Bhidayasiri R., Marti M.J., Maisonobe P., Om S. (2015). Factors influencing secondary non-response to botulinum toxin type A injections in cervical dystonia. Park. Relat. Disord..

[28] Jost W.H., Tatu L. (2015). Selection of Muscles for Botulinum Toxin Injections in Cervical Dystonia. Mov. Disord. Clin. Pract..

