# Supplementary material for: Does Ultrasound Guidance Improve the Effectiveness of Neurotoxin Injections in Patients with Cervical Dystonia? (A Prospective, Partially-Blinded, Clinical Study)
Source: Toxins (Basel). 2022 Sep 28;14(10):674. doi: 10.3390/toxins14100674 (PMC9610638; doi:10.3390/toxins14100674)
Supplement: Supplementary file 1 [file toxins-14-00674-s001.zip › toxins-1880524-Supplementary File S1-done.pdf]

toxins

MDPI

Supplementary Materials: Does Ultrasound Guidance Improve the Effectiveness of Neurotoxin Injections in Patients with Cervical Dystonia? (A Prospective, Partially-Blinded, Clinical Study)

Małgorzata Tyślerowicz, Jarosław Dulski, Justyna Gawryluk and Jarosław Sławek

The landmarks used for injections and muscle injection sites with US guidance

The landmarks used for the injections have been established as follows: sternocleidomastoid muscle in the upper third part (1cm depth); trapezius muscle at the junction of the neck and shoulder (1cm depth); splenius capitis at the level C2, along the line from C7 to mastoid (1cm depth); levator scapulae at the level C7, about 1cm ventral to the trapezius reference point (1cm depth); scalenus medius at the level C7, about 2 cm ventral to the trapezius (1cm depth); semispinalis capitis at the level C2, 2cm lateral to the spine (2cm depth); semispinalis cervicis at the lewel C5, in the posterior part of the neck, 2cm lateral to the spine (2,5cm depth); OCI at the level C2, 2cm lateral to the spine (3cm depth with aspiration).

Muscles with US control were injected as follows:

sternocleidomastoid muscle in the upper third part; trapezius muscle at the junction of the neck and shoulder; splenius capitis at the level C2 , along the line from C7 to mastoid ; levator scapulae at the level C7 in the middle part where it is uncovered by trapezius; scalenus medius at the level C7; semispinalis capitis at the level C2, 2cm lateral to the spine; semispinalis cervicis at the lewel C5, in the posterior part of the neck, 2cm lateral to the spine; OCI at the level C2, 2cm lateral to the spine . The depth of injection was determined by anatomical conditions.
