# Supplementary material for: Does Ultrasound Guidance Improve the Effectiveness of Neurotoxin Injections in Patients with Cervical Dystonia? (A Prospective, Partially-Blinded, Clinical Study)
Source: Toxins (Basel). 2022 Sep 28;14(10):674. doi: 10.3390/toxins14100674 (PMC9610638; doi:10.3390/toxins14100674)
Supplement: Supplementary file 1 [file toxins-14-00674-s001.zip › toxins-1880524-Supplementary File S2-done.pdf]

toxins

Supplementary Materials: Does Ultrasound Guidance Improve the Effectiveness of Neurotoxin Injections in Patients with Cervical Dystonia? (A Prospective, Partially-Blinded, Clinical Study)

Małgorzata Tyślerowicz, Jarosław Dulski, Justyna Gawryluk and Jarosław Sławek

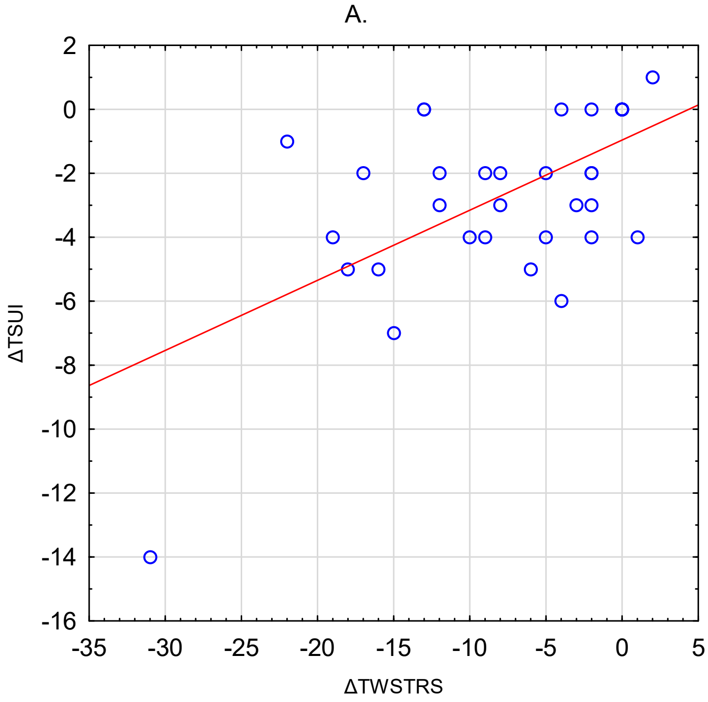

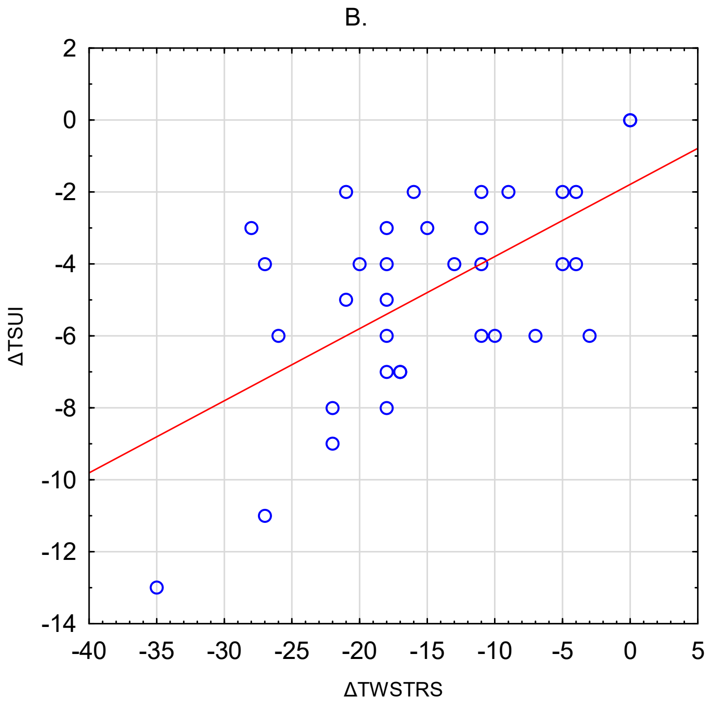
B.
ΔTSUI
ΔTWSTRS
2
0
-2
-4
-6
-8
-10
-12
-14
-40
-35
-30
-25
-20
-15
-10
-5
0
5

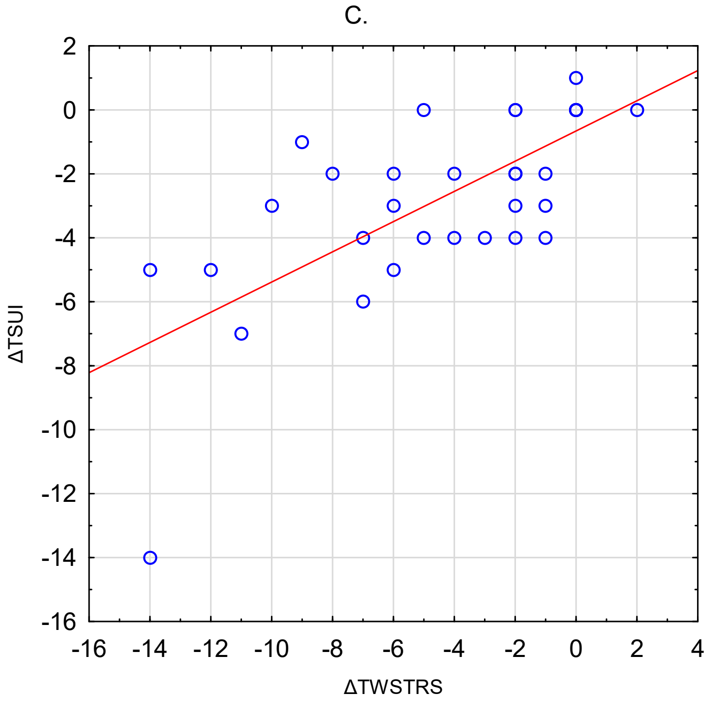
C.
ΔTSUI
ΔTWSTRS
2
0
-2
-4
-6
-8
-10
-12
-14
-16
-16
-14
-12
-10
-8
-6
-4
-2
0
2
4

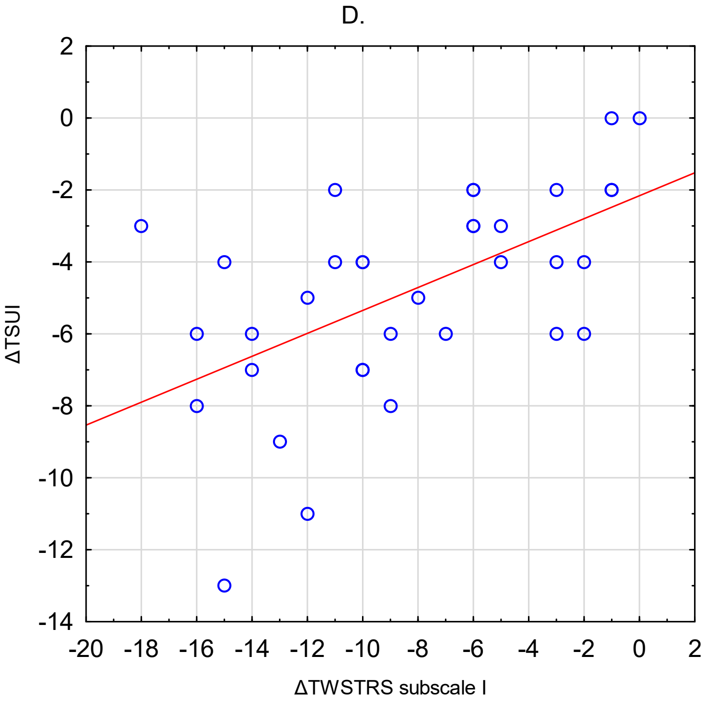

Spearman's correlation coefficients between ΔTWSTRS and ΔTSUI in patients with AL (A) and with US (B), and between ΔTWSTRS subscale I and ΔTSUI with AL (C) and with US (D).
